# K-EmoPhone: A Mobile and Wearable Dataset with *In-Situ* Emotion, Stress, and Attention Labels

**DOI:** 10.1038/s41597-023-02248-2

**Published:** 2023-06-02

**Authors:** Soowon Kang, Woohyeok Choi, Cheul Young Park, Narae Cha, Auk Kim, Ahsan Habib Khandoker, Leontios Hadjileontiadis, Heepyung Kim, Yong Jeong, Uichin Lee

**Affiliations:** 1 Korea Advanced Institute of Science and Technology, School of Computing, Daejeon, 34141 South Korea; 2 Korea Advanced Institute of Science and Technology, Information and Electronics Research Institute, Daejeon, 34141 South Korea; 3 Upstage AI Research, Yongin, 16942 South Korea; 4 LibL, Seoul, 06120 South Korea; 5 Kangwon National University, Department of Computer Science and Engineering, Chuncheon, 24341 South Korea; 6 Khalifa University of Science and Technology, Department of Biomedical Engineering, Abu Dhabi, 127788 United Arab Emirates; 7 Aristotle University of Thessaloniki, Department of Electrical and Computer Engineering, Thessaloniki, 54124 Greece; 8 Korea Advanced Institute of Science and Technology, KI for Health Science and Technology, Daejeon, 34141 South Korea; 9 Korea Advanced Institute of Science and Technology, Department of Bio and Brain Engineering, Daejeon, 34141 South Korea

**Keywords:** Human behaviour, Databases, Physiology

## Abstract

With the popularization of low-cost mobile and wearable sensors, several studies have used them to track and analyze mental well-being, productivity, and behavioral patterns. However, there is still a lack of open datasets collected in real-world contexts with affective and cognitive state labels such as emotion, stress, and attention; the lack of such datasets limits research advances in affective computing and human-computer interaction. This study presents *K-EmoPhone*, a real-world multimodal dataset collected from 77 students over seven days. This dataset contains (1) continuous probing of peripheral physiological signals and mobility data measured by commercial off-the-shelf devices, (2) context and interaction data collected from individuals’ smartphones, and (3) 5,582 self-reported affect states, including emotions, stress, attention, and task disturbance, acquired by the experience sampling method. We anticipate the dataset will contribute to advancements in affective computing, emotion intelligence technologies, and attention management based on mobile and wearable sensor data.

## Background & Summary

The proliferation of mobile and wearable devices has opened new avenues for research into understanding human beings using the data collected from these devices^1,2^. For example, studies have utilized ubiquitous sensors installed in various locations, such as in the human body, a vehicle, or a room, to understand diverse user behaviors and situations, including a user’s preference for shopping items^3^ and moments suitable for engaging in secondary tasks^4,5^. In addition, smartphones have been widely used as valuable data sources to detect stress^6^ and emotion^7^ or to analyze behavioral patterns relevant to various psychological disorders^8^ and states^9^.

A promising research area utilizing a data-driven approach to understanding humans is affective computing, which allows computing systems to recognize, analyze, and comprehend human emotions. An essential step for affective computing research is to obtain datasets associated with human affects; thus, researchers have made significant efforts to build datasets in various settings, as shown in Table 1. One typical setting in which datasets are collected is a controlled laboratory, where participants act out a specific affect or receive stimuli to induce specific affects; during this time, their physiological signals, facial expressions, and speech utterances are recorded. For example, studies have recorded emotional speeches by and facial expressions of professional actors^10,11^; additionally, audiovisual stimuli, such as emotional video clips, are often employed to cause participants to feel specific emotions and corresponding behavioral or physiological responses^12–20^. Furthermore, cognitively challenging tasks, such as preparing for a speech or debating sensitive topics, have often been used to induce stress or specific emotions in participants^17,21–23^. Although such a laboratory setting allows for acquiring a high-quality dataset, it lacks the ability to obtain real-world affects that people may experience in their daily lives.

**Table 1:** Comparison of the K-EmoPhone dataset with the existing studies (ECG: electrocardiogram; GSR: galvanic skin response; EEG: electroencephalogram; EMG: electromyograms; EOG: electrooculogram; HST: human skin temperature; PPG: photoplethysmography; MEG: magnetoencephalogram; RSP: respiration pattern; RRI: beat-to-beat R-R interval; n/s: not specified).

Study (year)	Data availability	Setting	# Participants	Collection period	Annotation					Data modality			
					Size	Strategy	Affect	Stress	Attention	Smartphone	Wearable	Other sensors	Survey
Emo-DB (2005)^10^	O	lab	10	n/s	535	acted	Ekman’s basic emotion^64^	—	—	—	—	audio	—
Haq *et al*. (2008)^11^	O	lab	4	n/s	480	acted	Ekman’s basic emotion^64^	—	—	—	—	video, audio	—
MAHNOB-HCI (2012)^12^	O	lab	27	27 minutes	540	induced	Ekman’s basic emotion^64^, one-item valence, one-item arousal, one-item dominance, one-item unpredictability	—	—	—	GSR, EEG, ECG, HST, RSP	video, audio, eye tracking	—
DEAP (2012)^13^	O	lab	32	40 minutes	1,280	induced	SAM^65^	—	—	—	GSR, EEG, EMG, EOG, HST, RSP, PPG	video	—
MoodScope (2013)^34^	X	field	32	2 months	n/s	signal (4/day), voluntary	one-item valence, one-item arousal	—	—	GPS, calls, app usage, messages, emails, web visits	—	—	—
StudentLife (2014)^25^	O	field	48	10 weeks	35,295	signal (8/day)	PAM^66^, one-item happiness, one-item sadness	one-item stress	one-item productivity	GPS, indoor location, Bluetooth, light, audio, activity, sleep, WiFi, acceleration, proximity, app usage, conversation, charging, screen	—	—	UCLA loneliness scale^67^, Flourishing scale^68^, academic performance, PHQ^47^, PSS^36^, BFI^27^
Bogomolov *et al*. (2014)^69^	X	field	111	7 months	n/s	interval (daily)	—	—	—	calls, messages, Bluetooth	—	weather	BFI^27^
cStress (2015)^21^	X	lab	19	1.5 hours	247	induced	—	Plarre’s subjective stress^70^	—	—	RSP, ECG, acceleration	—	—
		field	20	7 days	1,060	signal (15/day)							
SEED (2015)^14^	O	lab	15	2.5 hours	n/s	induced	one-item valence	—	—	—	EEG	eye tracking	
DECAF (2015)^15^	O	lab	30	2 hours	2,280	induced	one-item valence, one-item arousal, one-item dominance	—	—	—	—	MEG, EOG, ECG, EMG,video	—
Exler *et al*. (2016)^71^	X	field	6	4 weeks	1,821	interval (hourly), event (changes in calendar entries, etc), voluntary	shortend MDMQ^72^	—	—	cellular location, audio, app usage, messages, calls, light, connectivity, calendar entries, activity	ECG	—	—
DEAMER (2018)^16^	O	lab	23	60 minutes	414	induced	SAM^65^	—	—	—	EEG, ECG	—	—
MyTraces (2017)^35^	X	field	28	6 months	5,118	signal (20/day)	one-item valence, one-item arousal	one-item stress	—	notifications, app usage, screen, touch interaction, calls, messages, activity, GPS	—	weather	—
WESAD (2018)^17^	O	lab	15	2 hours	75	induced	PANAS^73^, SAM^65^	Plarre’s subjective stress^70^, SSSQ^74^, STAI^75^	—	—	ECG, GSR, EMG, HST, RSP, PPG, acceleration	—	—
Schmidt *et al*. (2019)^37^	X	field	11	16 days	1,083	signal (7.5/day), voluntary	SAM^65^	one-item stress, STAI^75^	—	—	GSR, PPG, acceleration, HST	-	PSQI^76^, PSS^36^
King (2019)^22^	X	lab	18	2 hours	144	induced		Plarre’s subjective stress^70^, PSS^36^, one-item stress	—	—	ECG, GSR	—	—
		field	18	2 days	100	signal (5/day)							
Tesserae (2019)^26^	O	field	757	56 days	n/s	interval (daily)	PANAS^73^	one-item stress, Davey’s one-item anxiety^77^	—	GPS, Bluetooth, light, audio, activity, sleep, acceleration, proximity, app usage, conversation, charging, screen, WiFi	PPG, step counts, stair counts, sleep, calories	social media usage, Bluetooth beacons	BFI^27^, STAI^75^, IRB^78^, ITP^79^, OCB-C^80^, OD^81^, AUDIT^82^, IPAQ^83^, PSQI^76^, GATS^84^, Shipley-2^85^
SEED-IV (2019)^18^	O	lab	15	3.6 hours	n/s	induced	Ekman’s basic emotion^64^, PANAS^73^	—	—	—	EEG	eye tracking	—
SEED-V (2019)^19^	O	lab	16	55 minutes	n/s	induced	Ekman’s basic emotion^64^	—	—	—	EEG	eye tracking	—
K-EmoCon (2020)^23^	O	lab	32	10 minutes	29,121	induced	one-item valence, one-item arousal, BROMP affect categories^86^	Plarre’s subjective stress^70^	—	—	PPG, GSR, EEG, ECG, HST, acceleration video, audio	—	
AMIGOS (2021)^20^	O	lab	40	23 minutes	38,642	induced	Ekman’s basic emotion^64^, SAM^65^, PANAS^73^	—	—	—	EEG, GSR, ECG	video, audio	BFI^27^
*K-EmoPhone* (2022)	O	field	77	7 days	5,582	signal (16/day), voluntary	one-item valence, one-item arousal, one-item emotion changes, one-item duration that a current emotion lasted	one-item stress	one-item attention, one-item task disturbance	GPS, battery, calls, WiFi, battery, connectivity, data traffic, ringer mode, screen, power, charging, activity, Bluetooth, media entries, messages	GSR, PPG, HST, RRI, acceleration, calories, step counts, ultraviolet, light	—	BFI^27^, PSS^36^, PHQ^47^, GHQ^49^

In recent years, there has been a growing interest in a new approach, the experience sampling method (ESM), which collects real-world data to overcome the limitations of laboratory data collection. This approach is often accompanied by a personal mobile device where participants are asked to respond to short questionnaires about their affects during their daily lives^24^. Depending on when the participants’ affects are sampled, ESM studies are divided into three categories: (1) interval-contingent sampling, in which participants’ responses are sampled at regular intervals (e.g., once a day); (2) signal-contingent sampling, in which participants’ responses are collected at random intervals; and (3) event-contingent sampling, in which prompts appear at the occurrence of a particular event. The collection of participants’ affects via ESM is often accompanied by the passive collection of sensor and interaction data from individuals’ smartphones and wearable devices. For example, the StudentLife dataset^25^ includes various data available on smartphones (e.g., ongoing physical activity, location, application usage, and ambient sound) annotated with affect labels (photographic affects and stress level) sampled eight times per day via ESM. In the Tesserae project^26^, researchers collected data from multiple modalities, including smartphones, wrist-worn sensors, Bluetooth beacons, and social media, and asked the participants to answer daily surveys on their affects, stress, and job performance.

Although significant efforts have been made to build datasets in affective computing, we believe there remains a need for real-world, multimodal open datasets containing various *in-situ* affect labels to help advance affective computing. In this study, we introduce *K-EmoPhone*, a real-world smartphone and wearable dataset with *in-situ* emotion, stress, and attention labels acquired from 77 students over seven days. This dataset aims to contribute to (1) understanding human affects with behavioral, contextual, and physiological data, (2) obtaining fine-grained affect states in terms of time, and (3) utilization in multiple domains, ranging from affective computing to attention management. We collected multimodal sensor data from the participants’ Android smartphones and Microsoft (MS) Band 2 smartwatches. In addition, we asked the participants to report their affect states, including valence, arousal, stress, attention, task disturbance, and emotional change, up to 16 times per day, either voluntarily or in response to prompts delivered via their smartphones. We hope this extensive dataset will contribute to a wide range of future research concerning data-driven human understanding.

## Methods

### Setup

**Fig. 1:**
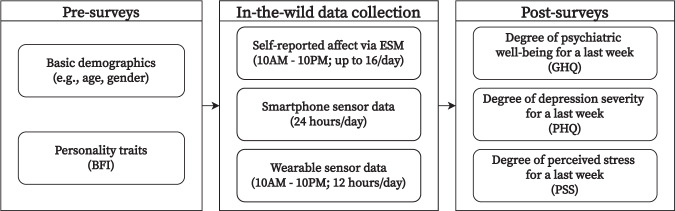
Data collection protocol.

The K-EmoPhone dataset aims to collect fine-grained *in-situ* affective and cognitive states, multimodal sensor data, and individual attributes relevant to personality and mental health. To this end, we conducted week-long real-world data collection accompanied by pre- and post-surveys (see Fig. 1).

#### Pre-surveys

Through pre-surveys, we obtained individual attributes that remained unchanged during the data collection period as follows:

- *Basic demographic information* included participants’ age and gender.
- *Big Five Inventory (BFI)* is a 44-item questionnaire that measures an individual’s disposition to five distinct personality traits: openness, conscientiousness, neuroticism, extroversion, and agreeableness^27^. These traits are known to persist over the long term^28^. We employed a shortened and Korean-translated version of the BFI, namely K-BFI-15. The K-BFI-15 comprises fifteen 5-point Likert-scale items with three items for each of the five personality traits, where summing the responses to items for each personality trait indicates a disposition toward that personality^29^. K-BFI-15 was developed based on an exploratory factor analysis of 720 participants’ responses to the Korean-translated version of the original BFI. It is known to be reliable for assessing five-factor personality domains, even with fewer question items than the original BFI, within the context of the Korean population and culture. One reason for using the K-BFI-15 instead of the original BFI was that the data collection was conducted in South Korea; thus, the participants were expected to be more fluent in Korean than in English. Another reason was to reduce the time required to respond to the questionnaire, thereby lowering the response burden on the participants.

#### Real-world data collection: *In-situ* emotion, stress, and attention

We employed smartphone-based ESM to capture various *in-situ* affective and cognitive states in real-world scenarios. To this end, we used PACO (https://www.pacoapp.com/), an open-source smartphone app that allows researchers to design and conduct ESM studies. During a 12-hour period within regular waking hours (i.e., 10 AM–10 PM), ESM prompts appeared on the participant’s smartphone as push notifications up to 16 times a day at random intervals (signal-contingent sampling). Each prompt was triggered every 45 minutes on average, and subsequent prompts were triggered at least 30 minutes apart. In addition, each prompt disappeared 10 minutes after its arrival if it was not answered to reduce recall bias because prior studies showed that excessive time lag between an ESM prompt and its response could compromise the ESM label quality^30,31^. We aimed to collect at least ten responses to the ESM prompts. However, there may have been cases where participants missed incoming prompts and thus could not respond before the expiration time (e.g., engaging in a formal meeting with a boss or taking a nap). Therefore, in addition to responding to the delivered prompts, we allowed the participants to answer our questionnaire voluntarily at any time.

For each prompt, participants responded to questions about emotion, stress, attention, task disturbance, changes in emotions, and the duration of the current emotion, as shown in Table 2. Because we intended to capture up to 16 responses daily, the primary concern in designing our questionnaire was to reduce the number of questions and thus relieve the participants’ response burdens. For example, in the case of emotions, we asked participants to independently report their valence levels (e.g., the degree of negativity or positivity of the emotion) and arousal (e.g., how calming or exciting the emotion is) using a 7-point Likert scale for collecting *in-situ* emotions. Alternatively, the Positive and Negative Affect Schedule (PANAS)^32^, which measures positive and negative affect levels, can be considered. However, the PANAS requires participants to respond to 20 items, which may be burdensome in real-world settings requiring frequent responses such as emotion collection via ESM. Instead, inspired by Russell’s circumplex model^33^, which represents different emotions using two-dimensional vectors of valence and arousal, emotion collection based on ESM has often asked participants to answer a single-item question for each emotional dimension, owing to several advantages^34,35^. One advantage is that participants are required to answer only two questions, thereby reducing their response burden; another is that valence-arousal interactions can describe various emotional states.

**Table 2:** Questions of the *in-situ* questionnaire (Q1: valence, Q2: arousal, Q3: attention, Q4: stress, Q5: emotion duration, Q6: task disturbance, Q7: emotion change).

*My emotion right before doing this survey was*			
Q1. very negative (−3)	~	very positive (+3)	[]
Q2. very calm (−3)	~	very excited (+3)	[]
*My attention level to my ongoing task right before doing this survey could be rated as*			
Q3. very bored (−3)	~	very engaged (+3)	[]
*My stress level right before doing this survey was*			
Q4. not stressed at all (−3)	~	very stressed (+3)	[]
*My emotion that I answered above has not changed for recent __ minutes*.			
Q5. [5, 10, 15, 20, 30, 60 min/I am not sure]			
*Answering this survey disturbed my ongoing task*			
Q6. not disturbed at all (−3)	~	very disturbed (+3)	[]
*How did your emotions change while you are answering the survey now?*			
Q7. I felt more negative (−3)	~	I felt more positive (+3)	[]

Like emotion assessment, our stress measurement methodology employed a single-item question asking participants to rate their perceived stress levels on a 7-point Likert scale instead of a multi-item questionnaire (e.g., the perceived stress scale or PSS, which consists of 10 items^36^) to lower response burdens on participants. Such a stress assessment with a single-item question has been widely used in real-world studies on stress assessment^22,25,26,35,37^.

In addition to emotions and stress, we considered measuring other affective and cognitive states because we intended our dataset to be used for several purposes. For example, we included two measures widely used in attention management (or interruptibility): the level of attention on an ongoing task immediately before the arrival of an ESM prompt^38–41^ and the level of task disturbance caused by responding to an ESM prompt^42^, both marked on a 7-point Likert scale. Furthermore, inspired by previous findings that responding to an ESM prompt can influence individuals’ affective and cognitive states (e.g., increase in stress^43^, annoyance^44^, and anxiety^45^), we clarified the extent to which answering a given ESM prompt caused participants’ emotions to be positive or negative on a 7-point Likert scale. Finally, we considered the duration of the current emotion in minutes, ranging from 5 to 60 minutes, which may be used to propagate emotion labels throughout the course of the response.

#### Real-world data collection: Multimodal sensor data

In addition to collecting affective and cognitive states via ESM, we recorded a wide range of sensor data from Android smartphones and the MS Band 2 smartwatches. For this, we implemented a special-purpose data collection software on an Android smartphone compatible with Android 6.0 or higher. This software unobtrusively collects sensor data reflecting mobility, network traffic, social communication, application usage, and device status 24 hours a day. In addition, our software was connected to MS Band 2 smartwatches via Bluetooth to obtain sensor readings relevant to physiological responses, environmental contexts, and mobility. Because the wireless transmission of a large amount of data would significantly consume the battery of MS Band 2, we collected data from MS Band 2 during the same period as our ESM schedule (i.e., 10 AM–10 PM; 12 hours) instead of 24 hours. The other time slots were intended to charge MS Band 2. Our software temporarily stores sensor data obtained from smartphones and smartwatches in the smartphone’s internal storage and uploads these data to our database server every hour.

Depending on the type of data, our data collection software collects sensor data using three sampling methods: (1) periodic, (2) adaptive, and (3) event-based sampling. During periodic sampling, sensor readings are collected at a predefined sampling rate (i.e., a sampling rate specified in the device catalog or manually set in the implementation of our data collection software). We note that the actual sampling rate can differ slightly from the predefined rate owing to I/O latency. Adaptive sampling dynamically adjusts the sampling rate, which typically depends on the OS policy of the device; for example, an Android smartphone varies its GPS sensor sampling rate according to its level of mobility. The operation of the GPS sensor is paused when no significant mobility is detected. When significant changes in the mobility of the device are detected, the GPS sensor is activated and records its location at a given sampling frequency. In event-based sampling, sensor readings are recorded only when subsequent readings differ. For example, a sensing modality indicating a smartphone’s ringer mode can only be present when users change it (e.g., from the vibrating to the silent mode). In such a case, some participants might keep their ringer mode the same and thus provide no readings in the ringer mode. More detailed information on each sensing modality, field, and sampling rate is presented later in *Data Records*.

#### Post-surveys

After a weeklong real-world data collection period, we conducted post-surveys to capture the individuals’ mental health. The detailed inventories are as follows:

- *Perceived Stress Scale (PSS)*, which consists of ten 5-point Likert-scale questions, is intended to measure the level of stress that an individual has recently experienced^36^. This study used the Korean version of the PSS, which has been proven valid and reliable for estimating perceived stress among Korean female workers^46^. The summing of all responses represents the total level of perceived stress, where a higher number indicates higher stress.
- *Patient Health Questionnaire (PHQ)* is used to assess the degree of depression over the last few weeks, which contains nine 4-point Likert-scale questions^47^. We used the Korean version of the PHQ, which has been proven reliable for assessing depressive symptoms in the Korean population^48^. Individual responses were transformed into a single severity score by summing.
- *General Health Questionnaire (GHQ)* was designed to measure the recent degree of severity of common psychiatric disorders^49^. Although the original GHQ has 60 questions, various shortened versions have been developed, such as 12-, 28-, and 30-item questionnaires, to assess psychiatric morbidity quickly. The most popular shortened version is the GHQ-12, which contains 12 questions. As we planned to recruit participants from the Korean population, this study employed the Korean-translated version of the GHQ-12, which has been proven reliable for measuring psychiatric disorders among Korean adults^50^. Responses to the 12 items are converted into a single severity score by calculating the sum.

We note that these inventories were originally intended to investigate recent mental health; thus, all question items in the inventories explicitly refer to a particular recent period (e.g., “*In the last month*, how often have you felt nervous and stressed?” in the PSS). Our post-survey aimed to measure mental health during the weeklong real-world data collection period. Therefore, we slightly modified the question items about mental health during that period (e.g., “*In the last week*, how often have you felt nervous and stressed?” in the PSS).

### Procedure

#### Ethics approval

Our study for building the K-EmoPhone dataset was approved by the Institutional Review Board (IRB) of the Korea Advanced Institute of Science and Technology (KH2018-42). We obtained written consent from participants who agreed to participate in this data collection after we explained the purpose of the K-EmoPhone dataset, detailed data collection procedures, data types we aimed to collect, possible risks caused by study participation (e.g., privacy leaks), and our countermeasures against such risks.

#### Data collection

Data collection was conducted from April 30 to May 8, 2019. We recruited 80 participants (24 females) with a mean age of 21.8 (SD = 3.8; range = 17–38) from our campus’s online bulletin board. They were all required to have smartphones whose Android OS version equaled or exceeded 6.0, on which our collection software could operate. Owing to the limited number of MS Band 2 smartwatches that we could provide, participants were assigned to three different collection periods, with each period lasting for a week (i.e., April 30 to May 7 for P29–P53, May 8 to May 15 for P01–P28, and May 16 to May 23 for P54–P80).

Each data collection period started with an hour-long offline introductory session to explain the study’s purposes, detailed procedures, possible risks caused by study participation (e.g., privacy leaks), and our countermeasures against such risks. Following the introductory session, participants who agreed to participate in this study were asked to sign a written consent form for study participation approved by our institution’s IRB. Participants then completed our pre-surveys, asking for basic demographic information (including age and gender) and their Big Five personality traits. Next, the participants were asked to install PACO and our data collection applications on their smartphones and were provided MS Band 2 smartwatches.

The real-world data collection began a day after the offline session and lasted for a week. During this period, we asked the participants to keep our applications active, secure the MS Band 2 on their non-dominant wrists from 10 AM to 10 PM daily, and report at least ten responses to ESM prompts in a day. Although we did not monitor the data collection progress of each participant in real-time, we instructed the participants to freely contact us if any problems occurred. After the real-world data collection period, our participants returned the MS Band 2 and uninstalled the applications installed for this study. The participants then completed the post-surveys to investigate their mental health over the previous week, using inventories such as the PSS, PHQ, and GHQ. We compensated each participant approximately 70 USD for participating in the data collection.

#### Data cleansing and privacy protection

After the real-world data collection, we initially collected 5,753 responses to *in-situ* questionnaires and 12.7 g of multimodal sensor data. However, through careful investigation, we found that data from three participants (P27, P59, and P65) had significant problems that could not be corrected. Such issues may have resulted from participants not adhering to our instructions or malfunctioning their smartphones’ data collection applications (i.e., PACO and our multimodal data collection application). In any case, we excluded the data collected from these participants. Detailed descriptions of these problems are provided below.

- *P27* generated a significantly larger amount of MS Band 2 data than other participants, with different sensor readings being recorded at the same timestamp. Because we could not confirm the correct reading among the different sensor readings at the same timestamp, we decided to exclude P27’s data.
- *P59* did not record any data that could be obtained from the smartphone. Because we wanted to build a dataset that included data from people’s smartphones and wearable sensors, we could not include P59’s data as they could not provide smartphone data.
- *P65* did not report any responses to the *in-situ* questionnaires. As a result, we could not investigate the affective and cognitive states using sensor data and thus excluded P59’s data.

As a result, our final dataset obtained from the remaining 77 participants (24 females) with a mean age of 21.9 (SD = 3.9; range = 17–38) included 5,582 responses to *in-situ* questionnaires and 11.7 g of multimodal sensor data. In addition, for data fields that may be used to identify participants, such as locations or phone numbers, we conducted preprocessing to conceal the obtained values (e.g., encryption, adding noise, value replacement)–a more detailed explanation on handling privacy-sensitive information is presented in *Data Records*.

## Data Records

**Fig. 2:**
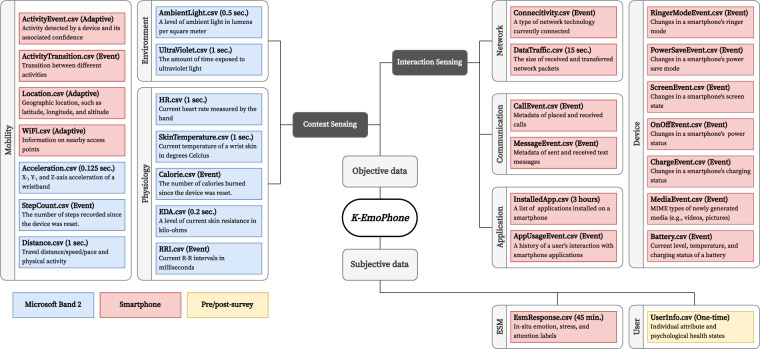
Overview of K-EmoPhone dataset.

The K-EmoPhone dataset^51^ is available at Zenodo (10.5281/zenodo.7606611). In the following sections, we present detailed descriptions of the K-EmoPhone dataset, including our participants’ characteristics and mental health obtained via pre- and post-surveys, self-reported *in-situ* affective and cognitive states sampled via the ESM, and multimodal sensor data from Android smartphones and MS Band 2 smartwatches. All data were formatted as CSV tables. Figure 2 presents an overview of the K-EmoPhone dataset.

### Individual characteristics and mental health

The demographic information, personality traits, and mental health of the participants are included in the following format:

- *UserInfo.csv*
  - pcode: a unisque identifier of each participant.
  - participationStartDate: the date when a participant started participating in the data collection
  - age: international age as of 2019.
  - gender: *M* and *F* if the participant is male and female, respectively.
  - openness: the personality trait indicating how accepting an individual is to intellectual curiosity, changes, and diversity, ranging from 3 to 15.
  - conscientiousness: the personality trait indicating how inclined an individual is to comply with social rules, expectations, and norms, ranging from 3 to 15.
  - neuroticism: the personality trait indicating how an individual exerts control over the external environment seeking mental stability, ranging from 3 to 15.
  - extraversion: the personality trait indicating how much an individual seeks a relationship, interaction, and attention from others, ranging from 3 to 15.
  - agreeableness: the personality trait indicating the extent to which an individual maintains comfortable and harmonious relationships with others, ranging from 3 to 15.
  - PSS: the degree of stress during the real-world data collection period as assessed by the PSS questionnaire, ranging from 0 to 40 (0–13: low; 14–26: moderate; 27–40: high).
  - PHQ: the depression severity as measured by the PHQ questionnaire, which ranges from 0 to 27 (0–4: minimal; 5–9: mild; 10–14: moderate; 15–19: moderately severe; 20–27: severe).
  - GHQ: the severity of common psychiatric disorders as measured by the GHQ questionnaire, ranging from 0 to 36 (11–12: typical; >15: evidence of distress; >20: severe problems and psychological distress).

### Self-reported ***in-situ*** affective and cognitive states

Participants’ responses to the *in-situ* questionnaires obtained via the ESM during the real-world data collection are included in the following format:

- *EsmResponse.csv*
  - pcode: a unique identifier of each participant.
  - responseTime: the Unix timestamp (i.e., milliseconds since Jan. 1, 1970, at UTC + 0) at which the participant completed a given questionnaire.
  - scheduledTime: the Unix timestamp at which an ESM prompt corresponding to a response appeared on the participant’s smartphone. This field was empty if the participant voluntarily submitted the response without any prompts or if the corresponding prompt expired.
  - valence: the degree of positive or negative feeling ranges from −3 (very negative) to 3 (very positive).
  - arousal: the degree of excitement ranges from −3 (very calm) to 3 (very excited).
  - attention: the level of attention to an ongoing task, which ranges from −3 (very bored) to 3 (very engaged).
  - stress: the level of stress ranging from −3 (not stressed at all) to +3 (very stressed).
  - duration: a measurement of how long the current emotion has lasted over the last few minutes, ranging from 5 to 60 minutes. This field can be empty if the participant answers, “I am not sure.”
  - disturbance: a level of how much an ongoing task was disturbed due to answering a given questionnaire, which ranges from −3 (not disturbed at all) to +3 (very disturbed)
  - change: the extent of emotional changes from answering the six questions above ranges from −3 (more negative) to 3 (more positive).

### Multimodal sensor data

Our multimodal sensor data collected from Android smartphones and MS Band 2 smartwatches during the real-world data collection are organized into subdirectories named according to each participant’s identifier (e.g., P##) under a parent directory named *Sensor*. For example, P01’s sensor data is placed in the *Sensor/P01* directory. Each subdirectory contains 27 CSV files, each representing a sensing modality. Every CSV file contains the field *timestamp* that indicates the Unix timestamp at which a given sensor reading was recorded. The following section elaborates on each sensing modality and its fields, excluding the field *timestamp*.

#### Android smartphone

- *Connectivity.csv*: the history of the connected network technology, which was recorded with event-based sampling only when the connectivity changes.
  - isConnected: *TRUE* if the network is connected, *FALSE* otherwise.
  - type: the type of the network technology whose values can be *MOBILE* (a typical mobile network), *WIFI* (WiFi), *VPN* (a virtual private network), *MOBILE_DUN* (a dial-up network), or *UNDEFINED* (disconnection).
- *DataTraffic.csv*: the history of the network data usage, which was recorded every 15 seconds.
  - rxKiloBytes: the size of the received data in kilobytes (kB).
  - txKiloBytes: the size of the transmitted data in kilobytes (kB).
- *CallEvent.csv*: the history of incoming/outgoing calls, which was recorded only when the participant made or received a phone call.
  - number: the contact’s phone number, which was encrypted with a one-way MD5 hashing except for the first four digits to prevent privacy leakage.
  - contact: the type of the contact, whose values are either: *MOBILE*, *HOME*, *OTHER*, *CUSTOM*, *WORK*, *MAIN*, *UNDEFINED*
  - timesContacted: the number of times that the contact previously communicated.
  - isPinned: *TRUE* if a shortcut is pinned, *FALSE* otherwise.
  - isStarred: *TRUE* if the contact is registered in the favorite list, *FALSE* otherwise.
  - duration: the time spent on this call in milliseconds (ms).
- *MessageEvent.csv*: the metadata of text messages (e.g., SMS and MMS), which was recorded only when the participant sent or received a text message.
  - number, contact, timesContacted, isPinned, isStarred: same as *CallEvent.csv*.
  - messageClass: the type of messaging service, either *SMS* or *MMS*.
  - messageBox: the message type, either *INBOX* (the message is received) or *SENT* (the message is sent).
- *AppUsageEvent.csv*: the history of participants’ interactions with smartphone applications. These data were collected with event-based sampling only when particular interaction events occurred.
  - name: the name of the application.
  - packageName: the unique identifier of the application
  - isSystemApp: *TRUE* if the application is a system app (i.e., the app is initially bundled as part of OS), *FALSE* otherwise.
  - isUpdatedSystemApp: *TRUE* if the application is an updated version of a system app, *FALSE* otherwise.
  - type: the type of interaction events whose values are either: *MOVE_TO_FOREGROUND* (the app moves to the foreground); *MOVE_TO_BACKGROUND* (the app moves to the background); *USER_INTERACTION* (the app interacts with the user in some way); *SCREEN_INTERACTIVE* (the app become available for interaction) *SCREEN_NON_INTERACTIVE* (the app become unavailable for interaction); *KEYGUARD_HIDDEN* (the keyguard has been hidden); *CONFIGURATION_CHANGE* (the device’s configuration has changed); *SHORTCUT_INVOCATION* (the app’s shortcut is selected by the user).
  - category: the category of the application. The values of this field were first retrieved from Google Play on May 28, 2022. For applications that disappeared from Google Play (e.g., deprecated applications), we found possible categories from application archive websites (i.e., https://apkcombo.com). The remainder of the applications that we could not find in any category were manually labeled as one of the existing categories. Possible values are one of 31 categories: *PERSONALIZATION*, *COMMUNICATION*, *PHOTOGRAPHY*, *SYSTEM*, *FINANCE*, *TOOLS*, *PRODUCTIVITY*, *HEALTH_AND_FITNESS*, *MISC*, *VIDEO_PLAYERS*, *TRAVEL_AND_LOCAL*, *MAPS_AND_NAVIGATION*, *LIFESTYLE*, *MUSIC_AND_AUDIO*, *HOUSE_AND_HOME*, *SOCIAL*, *ART_AND_DESIGN*, *GAME*, *SHOPPING*, *WEATHER*, *FOOD_AND_DRINK*, *EDUCATION*, *NEWS_AND_MAGAZINES*, *ENTERTAINMENT*, *SPORTS*, *BOOKS_AND_REFERENCE*, *BUSINESS*, *COMICS*, *LIBRARIES_AND_DEMO*, *BEAUTY*, *AUTO_AND_VEHICLES*
- *InstalledApp.csv*: the list of installed applications on the smartphone, which was recorded every three hours.
  - name, packageName, isSystemApp, isUpdatedSystemApp, category: same as *AppUsageEvent.csv*.
  - firstInstallTime: the Unix timestamp at which the application was first installed.
  - lastUpdateTime: the Unix timestamp at which the application was updated recently.
- *RingerModeEvent.csv*: the smartphone’s current ringer mode that was collected only when the ringer mode changed.
  - type: the currently-activated ringer mode whose values can be *NORMAL*, *VIBRATE*, or *SILENT*.
- *PowerSaveEvent.csv*: the smartphone’s current power-saving mode that was recorded only when the power-saving mode changed.
  - type: *ACTIVATE* if the power-saving mode is activated, *DEACTIVATE* otherwise.
- *ScreenEvent.csv*: the smartphone’s current screen states that were collected only whenever the screen state changed.
  - type: the current screen state whose values can be *ON* (the screen is turned on), *OFF* (the screen is turned off), or *UNLOCK* (the screen is unlocked).
- *OnOffEvent.csv*: the smartphone’s power state, which was recorded whenever the smartphone was turned on or off.
  - type: *ON* if the smartphone is turned on, *OFF* otherwise.
- *ChargeEvent.csv*: the smartphone’s charging state, which was collected when the smartphone is connected to or disconnected from the charger.
  - type: *CONNECTED* if the smartphone is charging, *DISCONNECTED* otherwise.
- *MediaEvent.csv*: the history of creating media, such as videos and photos, on your smartphone, which was collected when media were newly generated.
  - bucketDisplay: the bucket display name of the media (e.g., the name of the directory where the media is stored).
  - mimetype: the MIME type of the media
- *Battery.csv*: the status of the smartphone’s battery, which was collected when any change in the battery status occurred.
  - level: the battery’s percentage level (%).
  - temperature: the temperature of the battery in degrees Celsius (°C).
  - status: the current status of the battery that the Android OS displays in the system dialog, either *CHARGING* (the battery is started charging), *DISCHARGING* (the batter is discharging), *FULL* (the battery is fully charged), or *NOT_CHARGING* (the battery is not charging).
- *ActivityEvent.csv*: the history of physical activities detected by Google’s Activity Recognition API (https://developers.google.com/location-context/activity-recognition). These data were collected with adaptive sampling. For example, no reading was recorded if the smartphone became stable. Whereas, once the smartphone’s mobility was significantly changed, our implementation intended to collect these data every 15 seconds. Such a sampling rate could be varied by the smartphone’s status, such as if the power-saving mode was activated or the screen was turned off.
  - confidenceStill, confidenceWalking, confidenceRunning, confidenceOnFoot, confidenceInVehicle, confidenceOnBycicle, confidenceTilting, confidenceUnknown: the confidence level of activity detection associated with a particular activity, ranging from 0 to 1. The name after *confidence* indicates detected physical activities: *Still* (the device is stable), *Walking* (the device is on a user who is walking), *Running* (the device is on a user who is running), *OnFoot* (the device is on a user who is running or walking), *InVehicle* (the device travels by car), *OnBycicle* (the device is on a bicycle), *Tilting* (the device’s tilt relative to gravity has varied considerably), *Unknown* (no activity is recognized by the device).
- *ActivityTransition.csv*: the history of changes in detected physical activities, which was recorded when one activity transitions to another.
  - type: the transitional event of the physical activity, which was represented as the combination of the transition type (*ENTER*: the device is on a user who has started a certain physical activity; *EXIT*: the device detects that such physical activity is finished) and the physical activity type (*STILL*: the device is not moving; *WALKING*: the device is on a user who is walking; *RUNNING*: the device is on a user who is running; *IN_VEHICLE*: the device is in a vehicle); *ON_BICYCLE*: the device is on a bicycle), resulting in ten activity transition events. For example, *ENTER_WALKING* indicates that the user begins to walk.
- *Location.csv*: the history of locations visited. While our implementation asked the OS to report locations every three minutes or whenever a 5-meter displacement occurs, the actual sampling rate adaptively varied depending on the device’s mobility and battery level.
  - accuracy: the error bound of the recorded location in meters (m).
  - altitude: the altitude in meters (m).
  - longitude: the disguised longitude in degrees (°). Since the GPS coordinates are representative privacy-sensitive information and can be used to locate our participants, we disguised the actually-collected coordinates by adding a particular constant displacement to latitude and longitude. Because the relative spatial relationship between coordinates remains, such disguised coordinates would still be useful for the typical processing of location data, such as clustering, except for geocoding.
  - latitude: the disguised latitude of the GPS coordinate in degrees (°), which was processed in the same way as the longitude to protect possible privacy leakage.
  - speed: the movement speed measured by the smartphone overground in meters per second (m/s).
- *WiFi.csv*: This data is the list of nearby Wi-Fi access points (APs) scanned by the device. Because Our implementation tried to scan nearby APs every five minutes; however, the particular Android OS, whose version is equal to or greater than 8.0, allows the application to scan once in 30 minutes. In addition, the Android OS whose version equals to or is greater than 10 allows access to the information of APs scanned by other applications’ requests. Thus, the actual sampling rate can be highly different from five minutes.
  - bssid: the disguised MAC address of the detected access point. Since the MAC address can be used to locate our participants, we replaced original MAC addresses, represented as 48-bit hexadecimal digits, with 28-bit random numbers generated by a universally unique identifier (UUID), where each MAC address uniquely maps to one UUID number.
  - frequency: the band frequency of the detected access point in megahertz (MHz).
  - rssi: the received signal strength indicator in decibels per milliwatt (dBm).

#### MS Band 2 smartwatch

- *Acceleration.csv*: the acceleration of the wrist sampled at 8.
  - x: the acceleration of the x-axis in units of standard gravity (or G units), where 1 g is equivalent to 9.81 meters per second squared (m/s^2^).
  - y: the acceleration of the y-axis in G units.
  - z: the acceleration of the z-axis in G units.
- *StepCount.csv*: the number of steps that the participant has taken, which was collected at a sampling rate of 1Hz.
  - stepsToday: the total number of steps taken today.
  - totalSteps: the total number of steps taken since the participant has participated in the real-world data collection.
- *Distance.csv*: the participant’s mobility information sampled at 1Hz.
  - distanceToday: the total distance in centimeters (cm) that the participant has traveled today.
  - totalDistance: the total distance in centimeters (cm) that the participant has traveled since participating in the real-world data collection.
  - pace: the current pace in milliseconds per meter (ms/m).
  - speed: the current speed in centimeters per second (cm/s).
  - motionType: the type of physical activity detected by the device, whose values can be either *IDLE* (the device is stable), *WALKING* (the device is on a user who is walking), *JOGGING* (the device is on a user who is jogging), or *RUNNING* (the device is on a user who is running).
- *AmbientLight.csv*: the ambient brightness sampled at 2Hz.
  - brightness: the light intensity in lumen per square meter (lm^2^ or lx).
- *UltraViolet.csv*: the exposure of ultraviolet radiation, which was recorded every 60 seconds.
  - intensity: the current intensity index of the ultraviolet light, which is represented as one of *NONE* (a very low intensity), *LOW* (a low intensity), *MEDIUM* (a medium intensity), and *HIGH* (a high intensity).
  - exposureToday: the amount of time in milliseconds (ms) that the device has been exposed to the ultraviolet light today.
  - totalExposure: the amount of time in milliseconds (ms) that the device has been exposed to the ultraviolet light since the participant has participated in the real-world data collection.
- *HR.csv*: the participant’s heart rate, which was collected at a sampling rate of 1Hz.
  - bpm: the number of heartbeats per minute (b/min).
- *SkinTemperature.csv*: the skin temperature of the wrist sampled at 1Hz.
  - temperature: the skin temperature in degrees Celsius (°C).
- *Calorie.csv*: the number of calories that the participant has burned, which was collected at a sampling rate of 1Hz.
  - caloriesToday: the total number of kilocalories (kcal) burned today.
  - totalCalories: the total number of kilocalories (kcal) burned since the participant took part in the real-world data collection.
- *EDA.csv*: the participant’s skin resistance as measured by the electrodermal activity sensor, which was sampled at 5Hz.
  - resistance: the skin resistance measured in kiloohms (kΩ).
- *RRI.csv*: the interval between successive heartbeats, which was recorded only when consecutive readings were different.
  - interval: the time between the last two consecutive heartbeats in milliseconds (ms).

## Technical Validation

### Distribution of *in-situ* emotion, stress, and attention labels

During the real-world data collection, we collected 5,582 responses to *in-situ* questionnaires, where each participant provided 72.5 responses on average (SD = 16.0). In addition, 3,323 responses were received within 10 minutes of prompt arrival (mean = 43.7; SD = 19.4). The remaining responses were completed voluntarily or after a 10-minute expiration (mean = 29.3; SD = 16.3), where one participant (P71) never responded to ESM prompts and instead answered our questionnaires only in a voluntary manner.

**Fig. 3:**
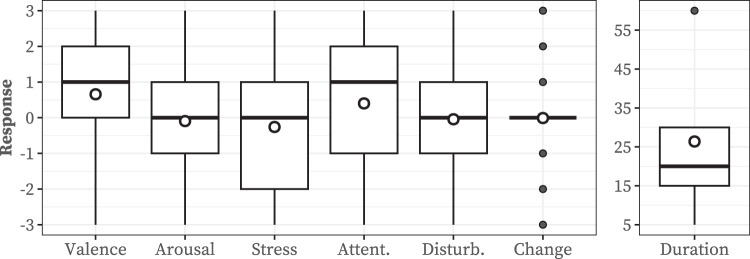
Distributions of responses to *in-situ* questionnaires. White dots are the mean of responses. Due to the difference in the scale, the *emotion duration* is illustrated in a separate sub-figure.

Figure 3 summarizes the responses to each question. Our participants reported a slightly positive level of valence (mean = 0.66; SD = 1.42) but a negligibly negative level of arousal (mean = −0.09; SD = 1.67). Additionally, they were slightly less stressed (mean = −0.26; SD = 1.62). Furthermore, their attention to the ongoing task was slightly positive (mean = 0.40; SD = 1.61). Responding to our *in-situ* questionnaire hardly disturbed their ongoing tasks (mean = −0.04; SD = 1.76) and barely changed their emotions (mean = −0.01; SD = 0.90). Their emotions lasted for 26.39 minutes on average (SD = 18.06).

Table 3 summarizes the repeated-measures correlation coefficients^52^ among the different response dimensions. Overall, there were statistically significant correlations across all dimensions. Arousal was positively correlated with valence (*r* = 0.386; *p* < 0.001) and attention (*r* = 0.435; *p* < 0.001) to a moderate extent, suggesting that participants were likely to focus on their ongoing task while feeling positive when they were emotionally aroused. However, the strong negative correlation (*r* = −0.591; *p* < 0.001) between valence and stress indicates that participants may feel negative when stressed. Interestingly, a negative (*r* = −0.222; *p* < 0.001) correlation between disturbance and emotional change implies that participants disturbed by responding to the questionnaire tended to start feeling bad.

**Table 3:** Correlation matrix among affect responses (*p < 0.05, **p < 0.01, ***p < 0.001).

	Valence	Arousal	Attention	Stress	Task disturbance
**Arousal**	0.386***				
**Attention**	0.288***	0.435***			
**Stress**	−0.592***	−0.202***	−0.152***		
**Task disturbance**	−0.029**	0.028**	0.118***	0.087***	
**Emotion change**	0.316***	0.167***	0.116***	−0.291***	−0.222***

The *Emotion duration* is excluded since it has non-numeric values.

### Machine-learning analysis

**Fig. 4:**
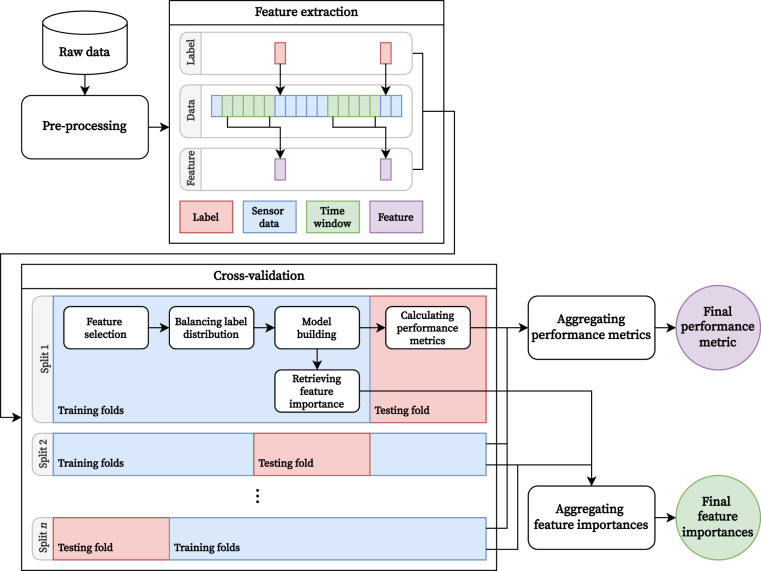
Our machine learning pipeline.

To confirm that our dataset is technically sound, we built and evaluated machine-learning models to predict individuals’ valence, arousal, stress, and task disturbance when an ESM prompt is triggered. For this, we preprocessed all data obtained from pre- and post-surveys and the real-world data collection. We then extracted many features corresponding to the responses to each *in-situ* questionnaire from the pre-processed data. These features and responses were used to build machine learning models such as XGBoost and Random Forest. We conducted a leave-one-subject-out (LOSO) cross-validation (CV) scheme to assess the generalizability of our models for an unseen user. In addition, we explored important features that significantly affected the structures of the models. The entire pipeline of our machine-learning analysis is illustrated in Fig. 4.

#### Preprocessing

Before building the machine-learning models, we first screened the data collected from the participants, considering the number of responses to *in-situ* questionnaires delivered via ESM prompts. Our task disturbance question aimed to measure how timely requests for answering questionnaires disturbed ongoing tasks. Therefore, responses obtained when participants reacted to delivered ESM prompts and responded to questionnaires before the expiration time (i.e., 10 minutes) would be in line with our purpose. For this reason, we first excluded 2,259 responses that were provided voluntarily or after a 10-minute expiration time. We then excluded 704 responses collected from 30 participants who reported fewer than 35 responses to ESM prompts, which is half the number of responses that we instructed (i.e., at least ten responses daily; a total of 70 responses for weeklong data collection). Consequently, we used 2,619 responses from 47 participants to build machine-learning models.

After screening, to simplify the classification task, we converted the 7-point Likert scale responses to the valence, arousal, stress, and task disturbance questions into binary classes by encoding responses greater than zero as *HIGH* and the remaining as *LOW*. The class distributions for each label were skewed to some extent: 1,556 (*HIGH*) vs. 1,063 (*LOW*) in valence; 1,033 (*HIGH*) vs. 1,586 (*LOW*) in arousal; 917 (*HIGH*) vs. 1,702 (*LOW*) in stress; 1,110 (*HIGH*) vs. 1,509 (*LOW*) in task disturbance.

In addition, we preprocessed multimodal sensor data to extract meaningful fields from each sensing modality and generate single-dimensional time-series sensor readings. For example, *Battery.csv* contains several fields that describe the current state of the smartphone’s battery, such as its temperature, level, and status. These fields were transformed into independent sensor readings. The entire list of the preprocessed data is presented in Table 4.

**Table 4:** Description of preprocessing on multimodal sensor data.

Raw data file	Preprocessed data	Data type	Description
**Smartphone data**			
ActivityEvent.csv	ACE_UNK	Num.	The ‘confidenceUnknown’ field.
	ACE_FOT	Num.	The ‘confidenceOnFoot’ field.
	ACE_WLK	Num.	The ‘confidenceWalking’ field.
	ACE_FOT	Num.	The ‘confidenceOnFoot’ field.
	ACE_VHC	Num.	The ‘confidenceInVehicle’ field.
	ACE_BCC	Num.	The ‘confidenceOnBicycle’ field.
	ACE_RUN	Num.	The ‘confidenceRunning’ field.
	ACE_TLT	Num.	The ‘confidenceTilting’ field.
ActivityTransition.csv	ACT	Cat.	The ‘type’ field of a physical activity that is currently conducted.
AppUsageEvent.csv	APP_PAC	Cat.	The ‘packageName’ field of an application that is currently in the foreground.
	APP_CAT	Cat.	The ‘category’ field of an application that is currently in the foreground.
Battery.csv	BAT_LEV	Num.	The ‘level’ field.
	BAT_STA	Cat.	The ‘status’ field.
	BAT_TMP	Num.	The ‘temperature’ field.
CallEvent.csv	CAE	Cat.	‘CALL’ if the phone is on the call; otherwise, ‘IDLE.’
ChargeEvent.csv	CHG	Cat.	The ‘type’ field.
Connectivity.csv	CON	Cat.	The ‘type’ field.
DataTraffic.csv	DAT_RCV	Num.	The ‘rxKiloBytes’ field
	DAT_SNT	Num.	The ‘txKiloBytes’ field.
InstalledApp.csv	WIF_COS	Num.	The Jaccard similarity between consecutive readings’ ‘packageName’ fields.
Location.csv	LOC_CLS	Cat.	7-bit geohash of the ‘latitude’ and ‘longitude’ fields.
	LOC_DST	Num.	Haversine distance in meters between consecutive readings’ ‘latitude’ and ‘longitude’ fields.
MediaEvent.csv	MED_VID	Num.	1 if the ‘mimetype’ field indicates a video file (i.e., video/*).
	MED_IMG	Num.	1 if the ‘mimetype’ field indicates an image file (i.e., image/*).
	MED_ALL	Num.	1 if the ‘mimetype’ field is not empty.
MessageEvent.csv	MSG_SNT	Num.	1 if the ‘messageBox’ field equals to ‘SENT.’
	MSG_RCV	Num.	1 if the ‘messageBox’ field equals to ‘INBOX.’
	MSG_ALL	Num.	1 if the ‘messageBox’ field is not empty.
OnOffEvent.csv	ONF	Cat.	The ‘type’ field.
PowerSaveEvent.csv	PWS	Cat.	The ‘type’ field.
RingerModeEvent.csv	RNG	Cat.	The ‘type’ field.
ScreenEvent.csv	SCR	Cat.	The ‘type’ field.
WiFi.csv	WIF_COS	Num.	The cosine similarity between consecutive instances’ ‘rssi’ fields.
	WIF_EUC	Num.	The Euclidean similarity between consecutive readings’ ‘rssi’ fields.
	WIF_EUC	Num.	The Manhattan similarity between consecutive readings’ ‘rssi’ fields.
	WIF_JAC	Num.	The Jaccard similarity between consecutive readings’ ‘bssid’ fields.
**Wearable data**			
Acceleration.csv	ACC_AXX	Num.	The ‘x’ field.
	ACC_AXY	Num.	The ‘y’ field.
	ACC_AXZ	Num.	The ‘z’ field.
	ACC_MAG	Num.	The square root of the sum of squared ‘x’, ‘y’, and ‘z’ fields.
AmbientLight.csv	AML	Num.	The ‘brightness’ field.
Calorie.csv	CAL	Num.	The difference between consecutive readings’ ‘totalCalories’ fields.
Distance.csv	DST_DST	Num.	The difference between consecutive readings’ ‘totalDistance’ fields.
	DST_MOT	Cat.	The ‘motionType’ field.
	DST_PAC	Num.	The ‘pace’ field.
	DST_SPD	Num.	The ‘speed’ field.
EDA.csv	EDA	Num.	The ‘resistance’ field.
HR.csv	HRT	Num.	The ‘bpm’ field.
RRI.csv	RRI	Num.	The ‘interval’ field.
SkinTemperature.csv	SKT	Num.	The ‘temperature’ field.
StepCount.csv	STP	Num.	The difference between consecutive readings’ ‘totalSteps’ fields.
UltraViolet.csv	ULV_INT	Cat.	The ‘intensity’ field.
	ULV_EXP	Num.	The difference between consecutive readings’ ‘totalExposure’ fields.

Cat.: categorical preprocessed data; Num.: numerical preprocessed data.

#### Feature extraction

For every timestamp at which participants completed *in-situ* questionnaires, we generated a total of 3,356 features from different data sources, including pre- and post-surveys, multimodal sensor data, and responses to *in-situ* questionnaires, as follows:

- From the pre- and post-surveys, we extracted 11 features that reflect basic demographics, personality traits, and mental health.
- From the preprocessed categorical sensor data, we generated 856 features that reflect the current sensor readings, the duration since the latest sensor readings changed, and the distribution of readings within a particular period just before participants reported their affective and cognitive states (i.e., a time window). Eight different sizes of time windows were considered: 30-second, 1-minute, 5-minute, 10-minute, 30-minute, 1-hour, 3-hour, and 6-hour.
- From the preprocessed numerical sensor data, we extracted 2,470 features relevant to the current sensor readings and the distribution of readings within a given time window. As in the categorical sensor data, we considered eight different time windows.
- From the *in-situ* questionnaires, we extracted 16 features relevant to the temporal contexts in which ESM prompts appeared on the participants’ smartphones. In addition, we generated three features reflecting the likelihood of a participant previously being in a *HIGH* affective or cognitive state within three different time windows, including 6-, 12-, and 24-hour. For example, for a given participant who reported their valence five times with three of these responses labeled as *HIGH* for 6 hours just before a particular timestamp, the feature value was 0.6.

A more detailed description of our features is presented in Table 5

**Table 5:** Description of extracted features in the technical validation.

Feature	Feature type	Description
**Pre- and post-surveys**		
PIF#AGE	Num.	The age of a participant.
PIF#GEN	Cat.	The gender of a participant.
PIF#BFI_OPN	Num.	The openness score in the BFI questionnaire.
PIF#BFI_CON	Num.	The conscientiousness score in the BFI questionnaire.
PIF#BFI_NEU	Num.	The neuroticism score in the BFI questionnaire.
PIF#BFI_EXT	Num.	The extroversion score in the BFI questionnaire.
PIF#BFI_AGR	Num.	The agreeableness score in the BFI questionnaire.
PIF#PSS	Num.	The degree of perceived stress score during the data collection period derived by the PSS questionnaire
PIF#PHQ	Num.	The degree of depression severity during the data collection period derived by the PHQ questionnaire
PIF#GHQ	Num.	The degree of psychiatric well-being during the data collection period derived by the GHQ questionnaire
**Pre-processed categorical sensor data (e.g., APP_CAT)**		
{DATA}#VAL = {VALUE}	Cat.	*TRUE* if the value recorded at the time nearest to a given timestamp is equals to ‘VALUE’, *FALSE* otherwise.
{DATA}#DSC	Num.	The duration between the latest value changes and a given timestamp.
{DATA}#DSC = {VALUE}	Num.	The duration between the time that a given ‘VALUE’ was recently recorded and a given timestamp.
{DATA}#ETP#{WINDOW}	Num.	The information entropy of readings within a given time window.
{DATA}#ASC#{WINDOW}	Num.	The number of changes between consecutive readings within a given time window.
{DATA}#DUR = {VALUE}#{WINDOW}	Num.	The duration that a ‘VALUE’ lasted within a given time window.
**Pre-processed numerical sensor data (e.g., DAT_RCV)**		
{DATA}#VAL	Num.	The value recorded at the time nearest to a given timestamp
{DATA}#AVG#{WINDOW}	Num.	The sample mean of data within a given time window.
{DATA}#STD#{WINDOW}	Num.	The sample standard deviation of data within a given time window.
{DATA}#SKW#{WINDOW}	Num.	The sample skewness deviation of data within a given time window.
{DATA}#KUR#{WINDOW}	Num.	The sample kurtosis deviation of data within a given time window.
{DATA}#ASC#{WINDOW}	Num.	The sum of absolute differences of data within a given time window.
{DATA}#BEP#{WINDOW}	Num.	The binned entropy of data within a given time window.
{DATA}#MED#{WINDOW}	Num.	The median of data within a given time window.
{DATA}#TSC#{WINDOW}	Num.	The time-series complexity estimate^87^ of data within a given time window.
***In-situ*** **questionnaires**		
ESM#DOW = {VALUE}	Cat.	*TRUE* if the day of the week when a given prompt was triggered equals ‘VALUE’ (which can be either *MON*: Monday; *TUE*: Tuesday; *WED*: Wednesday; *THU*: Thursday; *FRI*: Friday; *SAT*: Saturday or *SUN*: Sunday), *FALSE* otherwise.
ESM#WKD	Cat.	*TRUE* if the time when a participant received a given prompt is a weekend, *FALSE* otherwise.
ESM#HRM = {VALUE}	Cat.	*TRUE* if the name of the hour when a given prompt was delivered equals ‘VALUE’ (which can be either *DAWN*: 6AM–9AM; *MORNING*: 9AM–12PM; *AFTERNOON*: 12PM–3PM; *LATE_AFTERNOON*: 3PM–6PM; *EVENING*: 6PM–9PM; *NIGHT*: 9PM - 12AM; or *MIDNIGHT*: 12AM - 6AM), *FALSE* otherwise.
ESM#LIK#{WINDOW}	Num.	A prior likelihood of being in a *HIGH* affective state (i.e., the proportion of *HIGH* labels over whole labels within a given time window)

DATA: a name of preprocessed sensor data; VALUE: one of the possible values that a given categorical data can have; WINDOW: a name of a given time window, which can be either *S30* (30-second), *M01* (1-minute), *M05* (5-minute), *M10* (10-minute), *M30* (30-minute), *H01* (1-hour), *H03* (3-hour), *H06* (6-hour), *H12* (12-hour), or *H24* (24-hour); Cat.: a categorical feature; Num.: a numerical feature.

#### Cross-validation

We conducted LOSO CVs to approximate our models’ general performance in predicting the affective and cognitive states of an unseen user. For each participant, we partitioned our feature and label data into a testing fold with data from that participant and a training fold with data from the other participants (this set of the training and testing folds is hereafter referred to as “split”). We then trained our machine-learning models using the training fold data and evaluated them using the testing fold. As data from 47 participants remained after preprocessing, we repeated the partitioning, training, and evaluation processes 47 times.

For every training process, we first selected important features because the number of labeled data (2,619) was less than the dimensionality of our feature space (3,356), possibly leading to a *p* ≫ *n* (big-*p*, little-*n*) problem that requires more computing resources for model training and even deteriorates performance^53^. To this end, we trained an L1-norm support vector machine (the regularization parameter *C* was set to 0.01) with a linear kernel and squared-hinge loss function. This model estimates each feature’s coefficient, which indicates the effect of the feature on the prediction; the coefficients of the less important features become close to zero. Therefore, we selected only features with coefficients greater than zero. Note that we empirically selected the regularization parameter (i.e., *C*) that can reduce the feature space to about 10% of the number of labeled data (i.e., 2,619 to 261.9) because the rule-of-thumb on the number of samples required to build machine-learning models is unofficially known to be five or ten times the dimensionality of the feature space. Through feature selection, the mean dimensionality of the feature space per split was reduced from 3,356 to 235.6 (SD = 6.4) for valence, 245.9 (SD = 6.4) for arousal, 225.4 (SD = 6.6) for stress, and 209.5 (SD = 6.2) for task disturbance.

In addition, we balanced the label distribution on the training fold because our binary labels had skewed distributions, with ratios of *HIGH* to *LOW* being 1.46 for valence, 0.65 for arousal, 0.54 for stress, and 0.74 for task disturbance. Such an imbalance may cause machine-learning models to be less trained in the minority class, a class with smaller samples than other classes. To avoid this issue, we adjusted the ratio of HIGH to LOW to 1:1 by oversampling samples belonging to the minority class using the synthetic minority oversampling technique for data mixed with nominal and continuous fields (SMOTE-NC)^54^. We also considered the original imbalanced data to investigate the effects of oversampling on our models’ performance. Note that oversampling was conducted only in the training fold and not in the testing fold.

Subsequently, we trained the prediction models using two different learning algorithms: Random Forest^55^ and XGBoost^56^. Both algorithms are tree-based ensemble learning methods capable of handling a large feature space and capturing non-linear relationships between features. Because of this advantage, they have been widely used to predict user behaviors and cognitive states using mobile sensor data^57–59^, a setting similar to the K-EmoPhone dataset. We also trained a baseline model that always predicts the majority class for comparison with our models.

We then evaluated our prediction models using the testing fold data with performance metrics, including F1-scores for the minority and majority classes, the average of both F1-scores (i.e., macro-averaged F1-score), and accuracy. The final metric was derived by averaging the metrics calculated from 47 splits. Furthermore, the top ten important features for each split model were aggregated to further analyze the major contributing features of our models.

#### Prediction performance

Table 6 presents the performances for predicting valence, arousal, stress, and task disturbance across different learning algorithms and oversampling usages. Overall, the performance of our prediction models surpassed that of the baseline model in terms of the macro-averaged F1 and accuracy. Regarding the macro-averaged F1, the XGBoost algorithm performed better than the baseline and Random Forest, except for predicting arousal. However, the accuracy metric revealed that the Random Forest algorithm could better predict valence, stress, and task disturbance. Interestingly, oversampling improved our models’ performances in predicting the minority class, as shown in the F1 score concerning the minority class (e.g., F1*LOW* for valence and F1*HIGH* for the others).

**Table 6:** Performance evaluation results.

	Avg. F1 (SD)	F1_*LOW*_ (SD)	F1_*HIGH*_ (SD)	Accuracy (SD)
**Valence**				
Baseline	0.358 (0.114)	0.000 (0.000)	**0.715 (0.229)**	0.597 (0.233)
Random Forest (w/o oversampling)	0.523 (0.098)	0.358 (0.238)	0.687 (0.229)	**0.662 (0.115)**
Random Forest (w/ oversampling)	0.539 (0.093)	0.419 (0.236)	0.659 (0.238)	0.661 (0.115)
XGBoost (w/o oversampling)	**0.543 (0.104)**	0.408 (0.239)	0.677 (0.216)	0.659 (0.114)
XGBoost (w/ oversampling)	0.534 (0.097)	**0.428 (0.233)**	0.639 (0.216)	0.635 (0.109)
**Arousal**				
Baseline	0.364 (0.090)	**0.729 (0.180)**	0.000 (0.000)	0.600 (0.200)
Random Forest (w/o oversampling)	0.499 (0.087)	0.703 (0.173)	0.295 (0.181)	0.626 (0.132)
Random Forest (w/ oversampling)	**0.534 (0.096)**	0.670 (0.183)	0.399 (0.181)	0.623 (0.139)
XGBoost (w/o oversampling)	0.532 (0.084)	0.679 (0.177)	0.385 (0.209)	**0.634 (0.115)**
XGBoost (w/ oversampling)	0.529 (0.085)	0.626 (0.181)	**0.433 (0.187)**	0.600 (0.111)
**Stress**				
Baseline	0.390 (0.064)	**0.779 (0.129)**	0.000 (0.000)	0.655 (0.168)
Random Forest (w/o oversampling)	0.469 (0.076)	0.767 (0.131)	0.171 (0.172)	**0.666 (0.141)**
Random Forest (w/ oversampling)	0.508 (0.062)	0.730 (0.142)	0.285 (0.155)	0.644 (0.131)
XGBoost (w/o oversampling)	0.516 (0.058)	0.734 (0.135)	0.299 (0.187)	0.656 (0.111)
XGBoost (w/ oversampling)	**0.517 (0.073)**	0.685 (0.160)	**0.350 (0.173)**	0.620 (0.120)
**Task disturbance**				
Baseline	0.346 (0.136)	**0.692 (0.271)**	0.000 (0.000)	0.588 (0.294)
Random Forest (w/o oversampling)	0.517 (0.094)	0.661 (0.283)	0.372 (0.327)	0.722 (0.159)
Random Forest (w/ oversampling)	0.520 (0.081)	0.633 (0.316)	0.407 (0.317)	**0.727 (0.153)**
XGBoost (w/o oversampling)	0.523 (0.076)	0.626 (0.292)	0.420 (0.307)	0.708 (0.151)
XGBoost (w/ oversampling)	**0.525 (0.073)**	0.608 (0.280)	**0.442 (0.300)**	0.695 (0.155)

F1_*LOW*_ and F1_*HIGH*_ are the F1-scores when the labels *LOW* and *HIGH* are regarded as positive classes, respectively. Avg. F1 is the average of F1_*LOW*_ and F1_*HIGH*_ (i.e., macro-averaged F1-score). The best performance is highlighted in bold.

These results are notable compared to previous studies on using sensor data to predict emotional states. For example, the MAHNOB-HCI developed classification models that predicted three levels of valence and arousal based on physiological responses and eye gaze data collected in a laboratory setting^12^. The models built from peripheral physiological signals, including EDA, ECG, respiration patterns, and skin temperature, produced a macro-averaged F1 score of 0.39 for valence prediction and 0.38 for arousal prediction via LOSO CVs. Similarly, the DEAP evaluated binary classification models for valence and arousal with peripheral physiological responses obtained in a laboratory setting via LOSO CVs^13^, achieving a macro-averaged F1 score of 0.60 for valence prediction and 0.53 for arousal prediction. However, we trained prediction models with real-world multimodal data, where collecting high-quality sensor data is challenging. Therefore, it is noteworthy that our models showed comparable performance (0.53 for valence and 0.54 for arousal) to previous models with data collected in an in-lab setting, even though the MAHNOB-HCI tried to resolve more complicated classification tasks (i.e., multiclass classification) than ours (i.e., binary classification). Our results are also comparable to prior studies conducted in a real-world setting. For example, Schmidt *et al*.^37^ trained binary classification models for stress using physiological sensor readings collected from 11 participants over 16 days. These models achieved a macro-averaged F1 score of 0.47 for stress prediction via LOSO CVs, comparable to our models’ performance (i.e., 0.52). Consequently, we expect the K-EmoPhone dataset to have great potential for developing machine-learning models for emotion recognition, stress detection, and attention management.

#### Feature importance

**Fig. 5:**
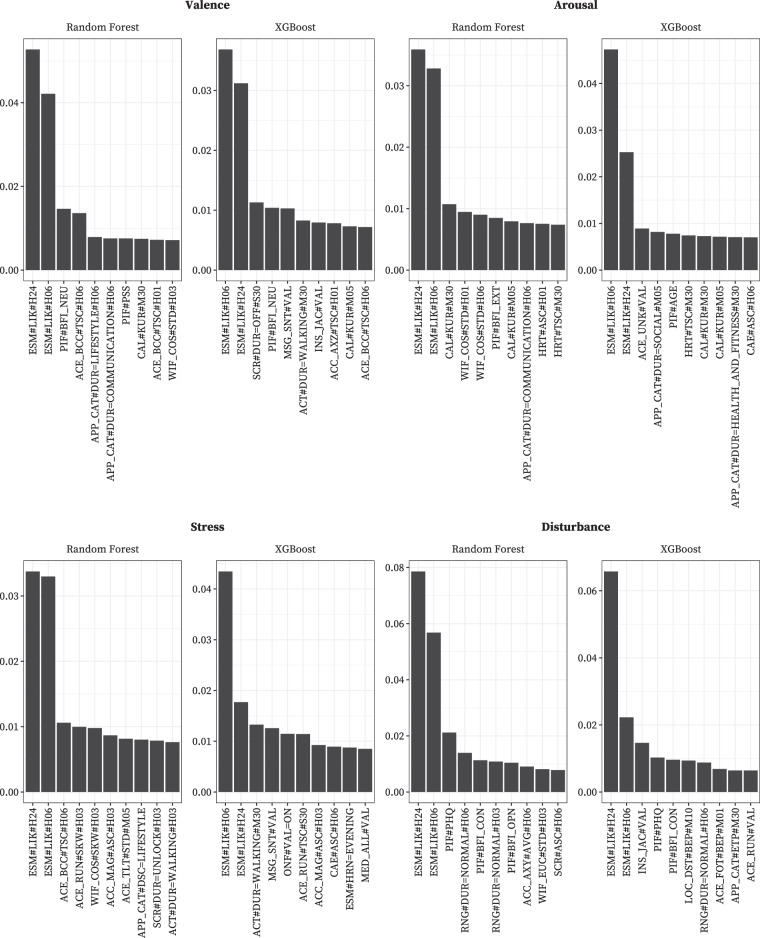
Top ten important features across models and labels.

We further analyzed the learning models to determine the major contributing features, as shown in Fig. 5. In general, the likelihood that affective or cognitive states were in a *HIGH* state within 6- and 24-hour windows before the arrival of a given ESM prompt (i.e., ESM#LIK#H06 and ESM#LIK#H24) was the most important feature for all prediction models, indicating that the affect in the last few hours significantly impacted the current affect. In addition, our models for predicting valence indicated that the neuroticism personality trait (i.e., PIF#BFI_NEU), which is relevant to emotional instability and sadness, is an important feature. Regarding arousal prediction, our Random Forest model indicated that the extraversion personality trait (i.e., PIF#BFI_EXT), which reflects excitability and emotional expressiveness, was significant. At the same time, XGBoost showed the duration for which participants used particular smartphone applications in the social category within a five-minute window (i.e., APP_CAT#DUR = SOCIAL#M05) was necessary. In predicting whether participants were stressed, the Random Forest and XGBoost algorithms considered how long participants walked within a 3-hour or 30-minute window before a prompt arrived as important (i.e., ACT#DUR = WALKING#H03 or ACT#DUR = WALKING#M30). Moreover, the task disturbance prediction was highly related to individuals’ depression severity and the conscientiousness personality trait, which is relevant to thoughtfulness (i.e., PIF#PHQ and PIF#BFI_CON).

## Usage Notes

### Potential applications

As discussed, the K-EmoPhone dataset is intended to help researchers understand affective and cognitive states using multimodal data, including physiological signals, individuals’ contexts and interactions captured by smartphones, personal attributes, and mental health. Our dataset provides several advantages with a large number of engaged participants (N = 77); a variety of sensing modalities reflecting mobility, behavioral, and smartphone usage contexts; timely response to affective and cognitive states; and real-world data collection settings.

We expect the K-EmoPhone dataset to help build machine learning models that predict mental well-being and productivity, such as emotion cognition^7,60^ and stress detection^21^. Additionally, this dataset can be utilized in attention management studies by considering attention and task disturbance levels^42,61^. Furthermore, with application usage and mobility information obtained from smartphones, this dataset allows researchers to investigate real-world behavioral patterns^8,9,62^. It is also promising to understand how emotional states can be affected by tasks that require timely responses to ESM prompts^63^.

### Limitation

Unfortunately, the MS Band 2 is no longer available for measuring physiological signals as the companion app stopped working on May 31, 2019. Commercial wrist-worn sensors from Fitbit, Garmin, Apple, and Empatica support sensing features similar to those of the MS Band 2; however, some sensing modalities are missing. Thus, researchers who wish to collect the same sensing modalities as those in the K-EmoPhone dataset may be required to consider two or more sensing devices. Nevertheless, we expect the K-EmoPhone dataset to be utilized as a first step toward exploring candidate sensing modalities for those studying affective computing with mobile sensors.

During the real-world data collection, we did not monitor the data collection process in real-time. While we provided detailed instructions of tasks that participants should consider (e.g., securing the MS Band 2 on their non-dominant wrist from 10 AM to 10 PM daily, reporting ten responses to *in-situ* questionnaires delivered via ESM prompts, and keeping our data collection application activated), there might exist a case where participants did not follow our instructions either intentionally or by mistake. Thus, the quality of the collected data may have been negatively affected in part. For example, as previously mentioned, one participant (P71) never responded to ESM prompts but consistently reported their affect voluntarily. Nonetheless, our technical validation shows that our dataset is promising for the binary classification of affective and cognitive states.

Our machine learning analysis binarized the labels with a simple threshold (i.e., zero), leading to an imbalanced label distribution. While we balanced the label distribution with oversampling during training and improved our models’ capability to predict the minority class, there may be other ways to address such an imbalance, with a greater potential performance improvement. For example, each participant may have their standards for rating their valence, which may result in responses from one participant being skewed toward a high valence and those from another toward a low valence. One possible way to handle this interpersonal difference is to set the threshold as the mean value of the responses for each participant instead of zero; in other words, responses higher than the per-person threshold may be encoded as *HIGH*, and those below the threshold encoded as *LOW*. We expect such a method to generate almost equally distributed labels, possibly improving performance without oversampling the minority class.

## Data Availability

We implemented an Android smartphone data collection application and used it to collect the K-EmoPhone dataset, which is available at https://github.com/Kaist-ICLab/K-EmoPhone_Logger. This application is intended to be run on smartphones with an Android API level of 21 or above. However, smartphones with an API level of 26 or above may not demonstrate the intended behavior owing to new privacy policies and deprecated data classes. In addition, our data exploration and machine-learning processes were written in a Jupyter notebook, which is available at https://github.com/Kaist-ICLab/K-EmoPhone_SupplementaryCodes.
